# ‘Please help me, I am so miserable!’: sexual health, emotions and counselling in teen and young adult problem pages in late 1980s Ireland

**DOI:** 10.1136/medhum-2021-012350

**Published:** 2022-10-25

**Authors:** Laura Kelly

**Affiliations:** School of Humanities, University of Strathclyde, Glasgow G4 0LT, UK

**Keywords:** History, Medical humanities, Sexual medicine

## Abstract

In 1984, the Irish Family Planning Association (IFPA) established a youth group comprised of young volunteers aged between 16 years and 20 years. The IFPA was responding to a perceived need for sexual health advice for young people in the absence of any formal sex education in Irish schools. The group established a telephone helpline and, from late 1987, was commissioned to provide advice columns for two Irish magazines for young people called *Hot Press* and *Fresh*. The advice columns run by the IFPA youth group provided an important educational and counselling service for young people on matters relating to sexual health and relationships. Letters to the two magazines also attest to a significant degree of ignorance around sexual and reproductive health among young people and the prevalence of risk taking with regard to contraception. Moreover, the letters were often frank and deeply honest about the feelings and emotions experienced by the letter writer. Replies to the letter writers from the IFPA group were sensitive and empathetic, but clear and unambiguous, highlighting the team’s approaches to sexual health counselling and ‘risk’ which were modelled on approaches by British groups such as the Samaritans, Grapevine and Brook. Drawing on the uncatalogued letters received by the youth group, as well as the published replies in the magazine advice columns, this article will shed light on the key sexual health concerns of young Irish people in the late 1980s. Ultimately, it will examine what these queries reveal about the impact of Ireland’s social and moral climate on teenagers’ emotional health. More broadly, the letters to these magazines illuminate the stigma, shame and silences around these issues in 1980s’ Ireland, and highlight the importance of magazines as a source of communication and an outlet for young people to express their emotions relating to sexuality, relationships and sexual health.

In the late 1980s, two new advice columns for young Irish people, ‘Tears ‘n’ Fears’ and ‘Sex Aid’ appeared in *Fresh* magazine (aimed at younger teenagers) and *Hot Press* magazine (aimed at teenagers and young adults), respectively. The letters received by these columns reveal much about the impact of Ireland’s social and moral climate on teenagers’ emotions around their sexual health, and how, in turn, advice columns acted as an informal method of counselling. Uniquely, these advice columns were organised by a group of young volunteers of the Irish Family Planning Association (later IFPA) Youth Group. An emotionally charged letter received by Sex Aid in November 1988 from a 17-year-old woman who was concerned she might be pregnant is typical of the dozens received by the two columns from young Irish adults struggling around issues of sexual health and sexuality in this period. This woman's account highlights the emotional impact of living in a sexually repressive environment and depicts the isolation she felt in navigating this situation. She explained that she was on the pill to regulate her periods and during sex with her boyfriend, the condom had come off inside her. She wrote:

I’m really depressed and thinking of suicide. We had a retreat at school and we touched on the subject of abortion. If I found out I was pregnant and if I committed suicide would this be an abortion. I’m Catholic and find it very hard to understand it. Please help me. P.S. I’m desperate and really worried. I live in a small town, gossip gets around very fast. (Letter from Jane 1988)

In a detailed reply published in *Hot Press* the following month the young woman was told that ‘the depression and worry you are going through at the moment is understandable but if you slow down, take it gently and deal with one problem at a time, everything should work out for you’. The young woman was advised that it was unlikely she was pregnant if she was taking the contraceptive pill as advised, but if she wanted to be certain, she could take a pregnancy test at her local family planning clinic. She was told that if the test proved positive, ‘don’t panic, it’s not the end of the world, by any means’ and she couldcontact the Adolescent Confidential Telephone Service (ACTS). (Sex Aid 1988)

In recent years, there have been a number of important studies which have expanded on our understandings of the history of sexual and reproductive health in the Irish context. (McAvoy 2012; Delay 2018 and Delay 2019; Earner-Byrne and Urquhart 2019; Ferriter 2012; Foley 2019). Moreover, while significant interventions by Eleanor O’Leary and Carole Holohan have shed light on youth culture in 1950s and 1960s Ireland respectively, we still know very little about young people’s experiences of sexual health in Ireland in the later twentieth century (Holohan 2018; O’Leary 2018). In the British context, scholars such as Caroline Rusterholz and Hannah Elizabeth have conducted valuable work in broadening our understandings of the controversies around young people’s sexual health and the development of sexual health services and education aimed at young people (Rusterholz (2021, 2021); Elizabeth 2021).

Concurrently, historians have also begun to use women’s magazines, and in particular, problem pages, to explore women’s experiences especially in relation to ‘private’ experiences of reproductive and sexual health (Clear 2016; Gudelunas 2017; Kelly 2021; Tinkler 2014). Tracey Loughran’s work, for instance, has shown how problem pages might allow the historian to uncover women’s experiences of infertility (Loughran 2017). Other scholars have argued for the value of problem pages as a primary source. In Adrian Bingham’s words, ‘far from being trivial, advice columns contributed to the process by which the British public became more self-reflexive about sex’ (Bingham 2012). While the historian should exercise care when using sources such as problem pages, as Clear notes, ‘they can, however, be taken with all due caution as representative of the problems that some people felt they could articulate and write about to a magazine and that a magazine could publish.’ (Clear 2016). This article aims to contribute to our knowledge of the sexual health experiences of young Irish men and women in the 1980s as well as recent scholarship which has used magazines in order to uncover individuals’ personal experiences in a variety of geographical contexts (Clear 2016; Gudelunas 2017; McRobbie 2000.)

For much of the twentieth century, Catholic ethos influenced government legislation surrounding sexuality and reproductive rights in Ireland. Sex outside marriage was strongly condemned and there was significant stigma towards unmarried motherhood, sex outside marriage and marriage breakdown in Ireland; 1984, the year that the IFPA youth group was established, was witness to two painful cases in Ireland which exemplify the impact of the sexually repressive environment. The first was the death of Ann Lovett, a 15-year-old schoolgirl, who died after giving birth to a stillborn baby at a grotto in Granard, County Longford, in January 1984 (Hug 1999). The second was the ‘Kerry Babies’ case, a 17-week inquiry after two newborn babies were found dead within 100 km of each other in April 1984 (McCafferty 1985). Moreover, the 1980s and early 1990s represented a period of significant backlash towards issues of sexual liberation, including divisive referendums over abortion (1983; 1992) and divorce (1986; 1995), and debate over the liberalisation of legislation relating to contraception (1985) (Ferriter 2012).

In the UK, comparatively, sex education in the twentieth century in the words of Lesley Hall ‘has not been a simple story of progress out of darkness and ignorance, into light and freedom’ (Hall 1995) and important work by scholars have noted significant controversies in the history of sex education provision in all parts of the UK (eg, Cook 2012; Davidson and Davis 2005; McCormick 2009; Pilcher 2005). It is also important to note that the provision of sex education in Northern Ireland was also deeply affected by social and religious conservatism. As Leanne McCormick has noted in relation to issues such as venereal disease, the provision of family planning services and sex education, for much of the twentieth century ‘the authorities were at pains not to offend the churches’. Writing in 2009, she remarked on how issues regarding sexual morality, including sex education, were ‘still overwhelmingly dominated by religious and moral rhetoric’ (McCormick 2009). The provision of sex education has also been affected by the nature of the schooling system in Northern Ireland where students were segregated along religious lines; it was not until the 1980s that integrated schools began to appear (Rolston, Schubotz, and Simpson 2005). As noted by Rolston, Schubotz and Simpson ‘the extent to which sex education in Northern Ireland is explicitly formed by religion is noteworthy and sets this society apart in significant ways from many other Western European societies.’ (Rolston, Schubotz, and Simpson 2005). Young men and women in 1980s’ Ireland were similarly limited—not only by lack of formal sex education—but by State and religious barriers to access to sexual health services and information. An article on sex education in Ireland by Frank Vaughan, a member of the IFPA Information and Education Department, highlighted the fact that

responsibility for both education and health, while nominally that of the State, was effectively handed to the Catholic hierarchy. The result of this is that the State funds Irish schools and hospitals that will only impart information or perform services that are in keeping with the Roman Catholic ethos. (Vaughan and O’Brien 1990)

This effectively meant that there was no national programme of sex education. However, Vaughan acknowledged that many young Irish people, while professing to be Roman Catholics, tended to ignore the Church’s teaching on sexuality. Yet, because of the Church’s control over sex education in the country, ‘young people are without access to information, services or decision making to prevent unplanned pregnancy’ (Vaughan and O’Brien 1990). A survey conducted by the IFPA in 1983 offers further evidence that young people were struggling to gain access to sexual health information. It revealed that 20% of visitors to IFPA clinics in 1983 were between the ages of 17 years and 20 years, and among these, 85% had been sexually active for an average of 1.8 years before seeking contraceptive advice, and more than 33% had been pregnant before coming to one of the clinics (O’Brien 1990). Mandatory sex education was not introduced into Irish schools until 1997 with the establishment of the Relationships and Sexuality Programme, a government initiative (Nolan 2018).

In addition, contraception had only been legalised in the country in 1979, with the introduction of the controversial Family Planning Act, described by Charles Haughey, then Minister for Health, as an ‘Irish solution to an Irish problem’, a disappointment to campaigners in favour and against legalisation (Beatty 2013; Girvin 2018). The act made contraception available for *bona fide* family planning purposes; this was widely interpreted as meaning that it was only available to married couples. In 1985, the law was liberalised under the Health (Family Planning) Amendment Act and non-medical contraceptives such as condoms and spermicides could now be bought by persons over the age of 18 years without a prescription at chemists, family planning clinics, maternity hospitals and Venereal Diseases (VD) clinics (Hug 1999). However, for sexually active teenagers, access to contraception remained difficult due to the age restrictions. In Niall Stokes’ introduction to an eight-page pull-out of key problems Sex Aid had received in the first 18 months of its existence, he wrote that the existing legislation meant that ‘those most in need of access to condoms – sexually active single people in their late teens and early twenties’ found them the most difficult to obtain. (Stokes 1989).

Moreover, the law criminalising homosexual acts had been in existence for over a century; many Irish men and women who wanted to live an openly gay life had left the country (Ferriter 2012). Political action on homosexuality was undertaken by campaigners such as Senator David Norris from the 1970s. Norris took a number of cases to the high court and supreme court in Ireland, ultimately culminating in a 1988 case in the European Court of Human Rights and the decriminalisation of homosexuality in 1993 (Hug 1999; Tiernan 2020). In addition, censorship in the 1980s meant that, in the words of Jon O’Brien, ‘even the simple books about how to have sex or how to use contraception were banned [under the Censorship of Publications Act].’ (O’Brien 2017).

This article draws on the (currently uncatalogued) archive of the IFPA which contains a wealth of material relating to the organisation’s work, particularly in the 1980s and 1990s.1 This archive includes the papers of the IFPA youth group which contains the original letters received by the Tears ‘n’ Fears and Sex Aid problem pages. Back issues of *Hot Press* and *Fresh* magazine have been consulted in order to view the responses to the problems which were not always retained in the archive. Authors of letters to the two magazines often prefaced their letters by emphasising a need for privacy and/or for their real name. Where names were attached to the letters, these names have been changed to avoid any possible identification. The archive includes 49 letters sent to the Tears ‘n’ Fears page and 65 letters sent to the Sex Aid page. Of the 49 letters to Tears ‘n’ Fears, 38 (78%) were from female correspondents and 11 (22%) from male correspondents, with this likely having a higher proportion of female writers because the magazine was largely aimed at young women. Of these, 34 gave addresses in Ireland (70%), 5 gave addresses in Northern Ireland (10%) and 10 did not give their address (20%). Of the 65 letters written to Sex Aid, however, 39 (60%) came from male correspondents and 26 (40%) from female correspondents; this likely reflects the broader readership of *Hot Press* which was a music magazine aimed at both men and women. The majority of letters to *Hot Press* came from addresses in Ireland, or anonymous authors; only three authors of letters gave Northern Irish addresses. Importantly, the gender breakdown of authors of letters highlights, perhaps unexpectedly, the high proportion of young men who used the magazines for information. We do not have exact figures for the readership of these two magazines, but in their media pack, *Fresh* estimated their target circulation in Ireland and Northern Ireland to be 36 500 with a projected readership of 250 000 people (47 500 in the 10–14 years age group and 2025 00 in the 15+ years age group). Fresh media pack, c 1987. In 1992, *Hot Press* estimated that their magazine’s circulation was 20 000 (Irish Press 1992).

Letters were answered by Jon O’Brien, as IFPA Youth Officer, primarily, but he consulted with other members of the youth group in devising his replies. Every letter was responded to personally by return of post and most of the letters received (barring those for which a request not to print them was included) were published in the magazine columns. All letters were published with the exception of letters which may have been suspected to be hoaxes, for example, a letter to Jody in 1987 from a 15-year-old at boarding school who claimed to have seen two nuns ‘naked, kissing and embracing each other’, and a letter to Sex Aid in 1988 where the author asked if ‘sexual intercourse with a sheep’ counted as losing his virginity. As we know, the voices of individuals in problem pages ‘do not appear unmediated in this form’ (Loughran 2020; Loughran, Mahoney, and Payling 2021). In the case of these two columns, letters were sometimes edited for length, but their appearance in the magazine was usually faithful to the content of the original letter. Marianne from Dublin, for instance, wrote to *Fresh* in July 1987, explaining ‘I am fifteen years old and I still have not got my periods. I have very small breasts and I’m very thin for my age. I am very worried. Could there be anything wrong with me? Could I be barren?’ Her letter was published unedited in the September 1987 edition of *Fresh* (Letter from Marianne 1987; Tears ‘n’ Fears 1987). Indeed, letters only tended to be edited if they were lengthy. For example, a letter from Susan which asked for advice about her friend who was ‘always talking about her weight and everything about her is wrong (in her eyes)’ was printed verbatim, with the end of the letter cut in the printed version for reasons of length (Letter from Susan 1987). Although the majority of letters published in the advice columns came from young people who wrote to the magazines, occasionally these were supplemented by devised problems which were based on calls to the telephone line or common questions that were asked by schoolchildren at sex education talks given by the IFPA. According to O’Brien, these supplementary letters were created ‘at the very beginning to get the ball rolling and then from time to time to cover areas of interest’. This helped to ensure that the advice columns covered areas where they knew concerns existed but also to ensure that the column did not become too repetitive from week to week (O’Brien 2021).

The letters to these two advice columns illuminate the stigma, shame and silences around sexuality in 1980s Ireland, the links between mental health and sexual health, and highlight the importance of magazines as a source of communication and an outlet for young people to express their emotions relating to sexuality, relationships and sexual health. In addition, this article seeks to emphasise the value in using sources such as teenage magazines in order to uncover young people’s experiences and encourages us to consider the value of less formal methods of counselling. Ultimately, this article will illustrate what these letters reveal about the impact of Ireland’s social and moral climate on teenagers’ emotions around their sexual health, and how, in turn, advice columns acted as an informal method of counselling.

I will also use frameworks from the history of emotions in my analysis of the letters, in particular drawing on the concepts of ‘emotional communities’, ‘emotional formation’ and ‘emotional frontiers’. Barbara Rosenwein describes emotional communities as being the same as social communities such as families, neighbourhoods and guilds, with the difference being that the ‘researcher looking at them seeks above all to uncover systems of feeling’ (Rosenwein 2002). Emotional formation, on the other hand, is a more recent concept devised by Vallgårda, Alexander and Olsen which refers to both a set of emotional structures organised in a particular pattern as well as ‘a process that depends on each individual learning the imparted codes of feeling.’ (Vallgårda, Alexander, and Olsen 2015). Emotional frontiers on the other hand, are the boundaries that exist between emotional formations, and represent the encounters young people have (‘from a minor misunderstanding to a seemingly insurmountable conflict’) which may be ‘particularly difficult to traverse’ (Vallgårda, Alexander, and Olsen 2015). This article asks: what can an analysis of letters to problem pages reveal about the emotions of historical actors, their sense of community or lack thereof, and the forces that affected their emotional formation, and the emotional frontiers they faced in relation to adolescent sexual health? And particularly, how did the Irish sociocultural context impact on young people’s experiences of sexuality and sexual health in the late 1980s and early 1990s?

## The IFPA Youth Group and Irish problem pages

The IFPA Youth Group had been established in 1984 and comprised young volunteers aged between 16 years and 20 years. Volunteers came from a variety of sources—some had been involved in IFPA work experience programmes, others came from schools and youth training programmes, while some were friends of relatives of IFPA staff or had been part of groups to whom the IFPA had given talks as part of its sex education work (O’Brien 1990). The IFPA by this stage had been in existence for 15 years, having started out in 1969 as the Fertility Guidance Company. The Fertility Guidance Company was the first group to openly challenge the Irish ban on contraception (in existence since 1935) by providing contraceptives to clients who paid a ‘donation’ rather than a fee; by 1973 the group had significantly expanded and was renamed the IFPA2 (see: Lindsey Earner-Byrne, 2010; McAvoy 2012). The IFPA youth group was founded by Christine Donaghy, then IFPA information and education officer who realised that there was a ‘need for a more direct and informal approach to young people…we believed that we needed to involve young people in their own education’ (Donaghy 2017). Following the completion of the training in October 1984, 8 of the original 14 members began the pilot project; 3 male and 5 female (O’Brien 1990). Volunteers self-selected whether they would remain on the programme after training (O’Brien 1990). Jon O’Brien, one of the original volunteers, was employed as a Youth Officer from 1985 and given responsibility for the youth group.

Over the course of its 10-year existence, the IFPA youth group ran a telephone line called the ACTS for young people, a sexual health clinic called the Young People’s Family Planning Centre, gave talks to groups of young people and schools, and most famously, were involved in a high-profile case at the Virgin Megastore in Dublin where members of the youth group illegally sold condoms in a record shop in order to challenge the law on the sale of contraceptives (Cloatre and Enright 2018). The ACTS was unique in that it was run by young people for young people, and received hundreds of phone calls—primarily from young men seeking advice on issues relating to sexual health. The fact that the majority of callers were male suggests that young Irish men were particularly affected by lack of access to sex education but also may imply that the anonymity offered by the phone service was particularly important to young men; young women may have been more likely to attain information from peers, for instance. In the Irish context, the work of ACTS was also unique in its targeting of young men and women; other telephone lines such as Tel-a-Friend (founded in 1974 by gay activists) were aimed at the LGBT community (McDonagh 2019).

From late 1987, as a result of their work on the ACTS, the IFPA youth group was commissioned to provide advice columns for *Hot Press* and *Fresh*. Moreover, in exchange for writing the advice columns, the ACTS was given free advertising. According to Jon O’Brien:

At a particular point in time, we needed to do more advertising to attract more young people to the telephone service. So we began writing to advice columns in a young teenage girls’ magazine called *Fresh* magazine. And in *Hot Press* we had a column every week called Sex Aid. In return for writing these advice columns we received free advertising from *Fresh* Magazine, and from Niall Stokes in *Hot Press*. Niall Stokes being very supportive of what we were trying to achieve. (O’Brien 2017)

*Fresh* was a popular music magazine which catered for those aged 12–15 years and the advice column Tears ‘n’ Fears was, according to a 1990 IFPA project report, ‘very broad based concerning typical teenage worries, such as physical appearance, exam pressure, parent problems, loneliness, as well as the usual sex related queries’ (O’Brien 1990). A ‘penpals page’ from 1987 in the magazine perhaps gives an indication of the interests of readers: most described themselves as music fans and listed their favourite bands. Elaine, for example, described herself as 15 years old and said ‘I’m mad into U2 and all the other promising Irish bands, also The Cure and Billy Idol’ (Tears ‘n’ Fears 1987). The first issue of the magazine appeared in July 1987 and the advice column ran for 2 years.

Because *Fresh* was particularly popular among young adolescent women, a female agony aunt pseudonym, ‘Jody Wilson’, was chosen. (O’Brien 1990). The use of a female persona for this agony aunt followed in the tradition of agony aunts being female and may have been selected in order to encourage young women to write, yet the fact that it was predominantly a man, O’Brien, behind the persona, is deeply unusual. And, in contrast to ‘traditional’ agony aunts, who generally tended not to have any training in counselling and drew moreso on their own experiences and views, the advice given by ‘Jody’ was coming from individuals, predominantly Jon O’Brien, who had received training in sexual health counselling (Loughran 2020). In order to further convince readers of ‘Jody’’s gender, a photograph of a young woman in her 20s, O’Brien’s then girlfriend, was used with her permission to represent the agony aunt. As a result of the popularity of the column, ‘Jody’ was asked to give the commencement address or lectures at girls’ schools and was also invited to do a live on-air television segment on the national television station Raidió Teilifís Éireann (RTÉ); because Jody’s real-life persona was a young man, it was not possible for Jon O’Brien to fulfil these requests. In the case of the television segment, Patricia Brennan, a member of the IFPA youth group appeared on the programme with advice from O’Brien on how to answer the questions (O’Brien 2021). In fact, in March 1989, the *Fresh* advice column was revamped ‘to reflect a male persona. No change was noted in the number or sex of letter writers’. The column was renamed ‘Dear Jon…’ and included a picture of Jon O’Brien who was described as ‘the new face in the anguish seat. He is a trained counsellor and is used to dealing with a wide variety of problems’ (O’Brien 1990).

*Hot Press* was a magazine founded by Niall Stokes, a young writer and journalist, in 1977. It was primarily a music magazine aimed at young adults and as well as including features on music, it also had feature articles on controversial topics such as abortion, AIDS and contraception. The magazine, according to IFPA youth group member John Callaghan, had a reputation for being ‘Very cool. Groovy. The music magazine’ (Callaghan 2017). The advice column in *Hot Press* was simply entitled ‘Sex Aid’ with the byline ‘Everything you ever wanted to know about sex and weren’t afraid to ask’ and most commonly addressed issues around sex and sexual and reproductive health. It ran from May 1987 until February 1990. According to an IFPA project report published in 1990, ‘Sex-Aid’, the *Hot Press* magazine’s advice column, conceived and written by the IFPA’s youth group, provided explicit and factual information on all aspects of sex and sexuality. Sex Aid prided itself on ‘telling it like it is’, not being coy and not giving way to the pressures to censor vital factual information’ (O’Brien 1990). It is clear, however, that the Sex Aid advice columns had a much wider scope than its target audience, suggesting that feelings of isolation and confusion around sexual health were not confined to teenagers and young adults. For example, Paul, a man, in his mid-30s, writing to Sex Aid in 1988 about a sexual problem, began his letter by stating ‘Thank you for publishing the letter and reply on ‘Mental Block About Sex’. I identified with the woman even though I am a man.’ (Letter from Paul 1988). Such letters highlight a clear absence of perceived community with whom problems could be shared and discussed.

These two magazines were following in an older tradition of Irish problem pages. As Carole Holohan has shown, the young people’s magazine *New Spotlight* dealt with issues relating to sexuality in its problem pages in the 1960s and 1970s, with responses to more sensitive issues being published in the ‘P.S.’ section without the original letter, while Caitriona Clear’s work has illuminated the role of Irish women’s magazines such as *Woman’s Way* which provided advice columns from the 1960s onwards (Clear 2016; Holohan 2018). However, *Fresh* and *Hot Press* were unique within this wider market of magazines in terms of their target audience of young people.

The name of the IFPA was directly associated with both of the advice columns which, according to a project report, ‘had obvious advantages in establishing the FPA as a helping organisation in the minds of the thousands of young people who read the magazine’. The IFPA youth group was also provided with advertising space in both publications for the ACTS which helped to reduce costs (O’Brien 1990). The advice columns run by the IFPA youth group provided an important educational and counselling service for young people and a source of advice for young people in the preinternet era who had very little access to information on matters relating to sexual health and relationships. While the ACTS was helping many young people with their problems, it is clear that some individuals did not feel comfortable speaking to a stranger on the phone, and the advice column therefore served an important function. For instance, a 17-year-old writing to the IFPA youth group stated ‘I could not get up the courage to phone so I taught (sic) I’d better write’(Letter from Anonymous 17 year old Undated) . Letters to the two magazines also attest to a significant degree of ignorance around sexual and reproductive health among young people and the prevalence of risk taking with regard to contraception. Moreover, the letters were often frank and deeply honest about the feelings and emotions experienced by the letter writer.

Replies to letters were sensitive and empathetic, but clear and unambiguous, highlighting the team’s approaches to sexual health counselling and risky behaviours such as unprotected safe sex. For example, a typical letter came from Louise, aged 18 years, in September 1987. She wrote to Sex Aid and explained that she had been going out with her boyfriend for almost a year, and ‘We feel we would like to have sex but don’t know enough about contraception. Could you tell about the pill and about condoms and which is better for us to use’ (Letter from Louise 1987). The published reply advised on the importance of using a method of contraception every time she and her partner had sex and provided clear, detailed information on both the condom and the pill and how these methods were used. Louise was encouraged to ‘go and have a chat with a sympathetic doctor or Family Planning Centre, (Hot Press 1987) where good advice will be available’ and to send for leaflets on other methods if she was interested from the IFPA (Sex Aid 1987a). This style of reply was modelled on approaches by British groups such as the Samaritans, Grapevine and Brook. In the Brook Advisory Centres, as Caroline Rusterholz’s article in this special issue shows, counsellors privileged ‘the notion of safety of intercourse over moral considerations about the status of the relationship’ (Rusterholz 2021). O’Brien’s style of replying to letters was also informed by the controversial straight-talking 1978 British sex education book *Make It Happy* by Jane Cousins. The book sold hundreds of thousands of copies in its first year of publication and aimed to deal honestly with topics relating to sex and sexuality. It had caused a furore in the UK, leading Conservative MP, George Gardiner, to put forward an amendment to the Government’s Education Bill which would have given parents the right to know what sex education was being provided in schools and the right to withdraw their children from sex education classes if they disapproved (The Times 1981).

The advice columns evidently created a space for young Irish people to discuss sexual health issues in an environment where such spaces were non-existent. As David Gudelunas has shown for the USA ‘not only do newspaper advice columns provide a space for conversations about topics that are prohibited elsewhere, but newspaper advice columns create this space within the pages of the daily newspaper’ (Gudelunas 2017). In addition, the *Fresh* and *Hot Press* advice columns also served an important educational function. As well as providing frank and clear information on contraception and other aspects of sexual health, the two advice columns also conveyed clear information about AIDS/HIV to young people during the 1980s. Kirra Minton’s recent article on Australian and American teen magazines in the 1980s has shown the important role that these magazines played in disseminating frank information on sex and sexual health during the AIDS era but also shows that these magazines provided teenage girls with a forum to ‘add their voices to the conversation around relationships, sexuality, responsibility, and AIDS, and know that they were being heard’ (Minton 2021). Similarly, Hannah Elizabeth, for example, has shown how in 1995 the British TV series Grange Hill addressed the HIV/AIDS crisis through five episodes. The programme produced ‘an idealised series of events wherein an AIDS-affected child could disclose her grief and fears, her disclosures made possible and advisable by sex education that had empowered her and her classmates’, ultimately iterating the need for AIDS education for young people (Elizabeth 2021).

In the Irish case, through their advice columns, the IFPA youth group also provided information on AIDS during a period when very little was being done in terms of public health efforts by the Irish government to educate people on the matter. In the absence of government-funded campaigns in the mid-1980s, it was gay activist groups such as Gay Health Action who had taken the lead in education campaigns (McDonagh 2021). As Páraic Kerrigan has shown, the Irish gay press, in particular *Out* magazine, was an important source of information on AIDS and the magazine was crucial in providing public information about AIDS, given that the Irish government did not take serious action in devising a public health campaign on the issue until 1993 (Kerrigan 2017). *Hot Press*, similarly, produced a number of informative and frank articles on the AIDS issue in the 1980s, at a time when there was significant silence from the government and hysteria in the popular media. For example, in February 1987, the magazine published an eight-page supplement entitled ‘AIDS: Don’t die of Hysteria’, which provided clear information on AIDS, as well as critiquing the Catholic Church and government responses to the virus and detailing the experiences of sex workers, drug users and gay men (Hot Press 1987). The Sex Aid column may be viewed as a significant element of this commitment to providing clear information on AIDS. According to *Hot Press* editor, Niall Stokes, the Sex Aid column was:

often determinedly, and unapologetically, explicit. There is, we feel, no room for ambivalence or evasion in a situation where ignorance can literally be dangerous, most specifically since the advent of AIDS. In this context, in particular, information and awareness are of absolutely vital importance – because to blunder on innocent of the perils associated with the lethal cocktail of intravenous drug abuse, multiple partners and unprotected sex can literally have fatal consequences (Stokes 1989).

The advice columns in *Fresh* and *Hot Press* were unafraid to address the issue of AIDS and received numerous letters around this topic. For instance, in November 1987, a letter from Peter (18) to *Fresh* stated:

I am a bit worried about A.I.D.S. I’m not a homosexual or anything like that but I know a guy and I think he is. We work together and I’d be afraid I’d get it from him. How can I protect myself. Should I tell the other guys in the job?

The reply from ‘Jody’ was very clear, explaining ‘No, you should not tell the other guys in your job. Why? Because you are totally wrong to make assumptions about your workmate and your comments show you are totally ignorant about A.I.D.S. A.I.D.S. is difficult to catch. There is absolutely no danger of getting it from daily contact at work, school or at home’. The reply went on to explain the key ways that AIDS could be transmitted and also dispelled myths that the disease could be ‘caught from cups, food, door handles, toilet or washing facilities or swimming pools’. It ended by emphasising that ‘Aids is not, and has never been, a gay (homosexual) disease. The sexual preference of your workmate is none of your business. Even if he is gay it does not mean that he has Aids. You are just as likely to catch the virus as anyone else if you practice ‘unsafe sex’ or share needles to inject drugs’. A number for an AIDS helpline was also provided for more information (Tears ‘n’ Fears 1987).

Letters also reveal the climate of fear around contracting the disease and the prevalence of risky sexual practices as well as the emotions attached to these. Emotions such as fear and terror were often attached to risky practices. A married woman writing to the magazine expressed her concerns that she was ‘terrified that I may have contacted (sic) Aids. I am experiencing a lot of the symptoms described on a video which I saw at a local meeting. As I may have contacted (sic) the disease from a man who is not my husband I am terrified to tell anyone. Please help’(Letter from Anoynmous married woman Undated). Similarly, Michael, aged 17 years, wrote to Sex Aid in 1987, ‘absolutely sick with worry’, about a sexual encounter with a man in his late 30s. While he wrote ‘Although I forced him to stop long before he or I climaxed, my penis came into casual contact with his anus, without actual penetration of course. I am a virgin, and have absolutely no knowledge of this man’s sexual history, and am petrified that I may have picked up the AIDS virus from him. His penis, however, only came into contact with my stomach. Is it possible that I may have put myself at risk. Please help’. (Sex Aid 1987). Letters about AIDS convey significant ignorance about how the disease could be transmitted. A letter written to Sex Aid in January 1989, for example, asked:

Recently, myself and some friends were messing around with a used condom we found, throwing it at each other. It was only recently used as it looked clean and was well lubricated. Afterwards we had chips and burgers. I know that I licked my fingers, and I had not washed my hands. I know it’s stupid, but I’m a little worried about contracting AIDS or some venerial [sic] disease from it. Could you put my mind at rest?’ The letter also asked whether the AIDS virus could be killed in the presence of sunlight, wondering ‘if this is true, then surely drug abusers could share needles in safety if they exposed the syringe to sunlight???’ (Letter from Anonymous 1989).

Replies to letters such as this one therefore attempted to dispel common myths about the way the disease could be transmitted. The letter was printed in *Hot Press* and the reply assured the reader that ‘the risk of transmission of HIV infection by contact with a condom is slight to non-existent. No cases have been documented as having been transmitted in this way and from the level of contact you have described you have no need to worry’ and went on to refute the idea about the virus being killed in the presence of sunlight (Sex Aid 1989). In the absence of adequate government public health campaigns around AIDS, advice columns such as Sex Aid and Tears ‘n’ Fears were clearly a vital source of information for Irish young people and helped to complement the work of Irish activist groups such as Gay Health Action.

## Emotions and sexual health

Georgia Carr and Monica Bednarek’s analysis of problem pages in *Dolly* magazine in the 1990s and 2010s highlighted that the 1990s keywords tended to relate to sexual health, whereas the 2010s keywords were more likely to relate to emotions and mental health, suggesting ‘a preoccupation with sexual health in the 1990s, which has shifted over time to a preoccupation with mental health and emotions more generally in the 2010s’ (Carr and Bednarek 2019). I would argue, however, that these two issues, sexual health and mental health, are not mutually exclusive, and that the letters to Irish problem pages in fact indicate that young people often linked sexual health to emotional well-being or mental health issues. Indeed, from a history of emotions perspective, the letters received by the problem pages are a rich historical source which convey much about the emotions that resulted from young people’s concerns around sexual health in 1980s’ Ireland and how these were shaped by Ireland’s social and cultural climate. Or, as Claire Langhamer has argued while ‘It is easy to dismiss magazine problem pages as straightforward exercises in emotional prescription and vehicles for the assertion of authority’, when columns are read against the grain, they ‘offer a more complex and contested view’ of emotion dialogue (Langhamer 2013).

It was not uncommon for young adults and teenagers, who wrote to the magazines for advice on sexuality and sexual health to express intense feelings of loneliness, shame and depression, and in some cases a concern that their problem might not be taken seriously. One 16-year-old girl writing to Jody at Tears ‘n’ Fears for advice, for example, stated ‘Please print my letter and answer it before I find the courage to kill myself and make the world a better place (no joke)’ (Letter from Anonymous 16 year old girl). Likewise, 19-year-old Simon wrote to Jody that he was worried about never having had a girlfriend and how he looked, enclosed a photo of himself ‘to ask for your *honest* opinion’. He also wrote ‘P.S. My problem may not sound much to you – but I am *so* depressed. Please print my letter (but not the photo)’ (Letter from Simon). These two examples clearly indicate the significant emotional impact that concerns around sexual health issues and appearance had on individuals’ mental health. Authors of such letters were unafraid to express these emotions in writing but their letters suggest that they perceived a lack of community with whom they could frankly discuss these issues.

Yet, it is important to note that given the overwhelming atmosphere of stigma and shame around sex in Ireland, writing to the magazine should also be viewed as a powerful act of agency on behalf of the young letter writers and likely required some bravery to compose such letters and traverse these ‘emotional frontiers’. The gravity of this act is also reflected in the fact that many young people felt the need to apologise for writing into the column. For example, a young woman, Niamh, writing to Sex Aid in 1989 ended her letter stating ‘Sorry for being so cheeky but it took courage to write like this’ (Letter from Niamh 1989). Another writer in 1989 opened her query with ‘I’m sorry to bother you about this but I really don’t know where else to turn’. Similarly, ‘Erasure fan’, aged 20 years, writing to Sex Aid in 1989, about his feelings of depression around his sexuality, added a note of ‘P.S. Sorry about the long letter but I needed to write down all my feelings on paper’ (Letter from Erasure Fan 1989).

Letters also reveal much about the forces which shaped the emotional formation of correspondents. As Vallgårda, Alexander and Olsen have suggested, ‘Emotional formations, while distinguished by certain overall hierarchies of feeling, also tend to be characterised by a high degree of diversity across space, class, ethnicity, age and gender.’ (Vallgårda, Alexander, and Olsen 2015). In the Irish context, lack of formal sex education clearly had an impact on young people’s attitudes to sex and associated emotions. For instance, Niall, aged 18 years, writing to Tears ‘n’ Fears in 1987 explained the impact that lack of sex education had on him:

Sex is something we never talked about at school beyond finding one or two things about plants and frogs which was no help to me at all. I’ve tried on numerous occasions to ask my parents questions but I’ve never got any answers I could make sense of. I have no brothers and sisters and my friends seem to be even more confused than I am. I need to know about these things, not because I want to have sex now, but because I feel ignorant. I also get worried sometimes that my body is not normal.

In ‘her’ reply, Jody encouraged Niall to ask his parents for advice, and if they continued to be reticent on the issue, to phone the ACTS to ask them about anything he was worried about (Tears ‘n’ Fears 1988). If we use the concept of ‘emotional formation’ here, the silences around sex education in both school and home clearly contributed to Niall’s sense of confusion and anxiety around his body. He is clearly part of an emotional community of peers which also lacks any understanding of sex, and trying to gain an understanding of how his body works may be seen to be an ’emotional frontier’ that he has to traverse.

Emotions also became pronounced in issues around young people’s sexual health as a result of the risks surrounding unintended pregnancy, the risk of STDs such as AIDS, and related concerns around privacy within individuals’ communities and lack of access to sex education. Moreover, the letters received by the problem pages provide insights into the impact that Catholic teachings had on individuals’ emotional formation, particularly in relation to attitudes to sex, and how the conservative moral climate and pervading culture of stigma and shame may have amplified these problems. Shame around sex, which would have been indoctrinated through Catholic teachings, meant that many letter writers expressed feelings of fear and embarrassment. David, 18, was concerned about one of his testicles being larger than the other, wrote in a letter to Sex Aid ‘I have tried to pluck up the courage to see a doctor but cannot’ (Letter from David). David’s testimony here highlights the impact of emotions such as fear on accessing sexual health services. Others blamed their religious upbringing for their problems. Rich, aged 22 years, put his sexual problems partly down to the fact that ‘I was very religious ever since I can remember, not in a bigoted way, I thought if I saved myself I would find somebody I loved and everything would be alright’.(Letter from Rich, 1987).

Young Irish people evidently had significant emotional frontiers to overcome in relation to sexual health. Disparities in access to contraception meant that some young people were engaging in risky behaviours such as unprotected sex. Illustrating this point, the introduction to a pull-out edition of Sex Aid in March 1989, stated, ‘While the vast majority of 16 and 19 year olds are – to one degree or another – sexually active, it is only the rare exceptions who are yet possessed of the audacity to step up to the chemists counter in a small town and ask for a couple of packets of Durex. And so Irish teenagers are forced to continue to shoot in the dark…’ (Stokes 1989). A letter to Sex Aid in 1987 from a young woman also highlights the challenges that people faced in accessing contraception and the gendering of responsibility for contraception. The author of the letter was planning to sleep with her boyfriend for the first time and explained:

We got some condoms off a friend, only Mark’s not too sure how to use them. We don’t have the box with the instructions on it – how should he put one on? – Somebody said to me that it’s not the girl’s job to be carrying condoms, it’s up to the boy to supply them. Do I leave it to Mark, then, to get them? He’s very shy about asking at the chemist’s for them’. (Letter from Sandra 1987)

In their reply to the young woman’s letter, Sex Aid advised that ‘both partners have a responsibility to ensure that contraception is used each and everytime they have sex (unless of course, you’re planning to have a baby!) Carrying condoms can be seen simply as part of developing a mature attitude towards sex which is something we all need to do’. The woman was advised to purchase condoms from a family planning clinic because ‘you can be absolutely sure that they are stocked there’ and suggested that they should go to buy them together, or ‘if your boyfriend refuses to go, then get them yourself. Whatever you feel about his reluctance, you owe yourself the protection that condoms offer’ (Sex Aid 1987).

Letters to the magazine also highlight the problems that lack of access to contraception caused for young people and the emotional distress that could result. A 13-year-old wrote to Jody stating ‘I often worrie [sic] about getting pregnant for I know that a condom does not give 100% assurance against pregnancy. Is it possible to get a contraseptive [sic] pill without being over 16 and needing parents [sic] permission’. The letter does not appear to have been published in the magazine, potentially due to fears that this was an attempt at entrapment into breaking the law by conservative campaigners. Indeed, with the ACTS telephone line, oral history respondents from the youth group expressed their concerns about this issue and a sense that they had to be careful with the advice they provided, for fear of prosecution. Fears of entrapment had been heightened following an injunction granted to the Society for the Protection of Unborn Children (SPUC) by the High Court in December 1986, against two pregnancy counselling services, Open Door Counselling and the Dublin Well Woman Centre (founded in June 1983). This meant that Open Door Counselling was forced to shut down, and the Dublin Well Woman Centre had to suspend its counselling services (Barry 1988). And, in 1973, the IFPA and another family planning clinic, Family Planning Services, had been taken to court after a conservative campaigner, John O’Reilly, wrote a letter to Family Planning Services with a postal order for 75 pence requesting condoms and had his 11-year-old daughter Deirdre sign it. Family Planning Services sent back condoms in a plain envelope addressed to his daughter. In the court case that followed at Dublin District Court, the charges were dismissed (Irish Press 1974). Members of the ACTS group were therefore operating within a climate of fear around sexual health issues.

In particular, letters from queer men and women illustrate the significant distress caused by individuals trying to address their queerness in 1980s’ Ireland. For example, in 1988, a young man in his early 20s wrote to Sex Aid; at the top of the letter he included a note ‘This letter is *genuine*’. Beginning his letter, he explained ‘What I’m about to tell you has been troubling me for a long time. I am in my early 20s, male, and I am confused, very lonely and depressed’. The man went onto explain about how he had been in love with his best friend when they were teenagers but had ended the friendship abruptly to avoid confronting his feelings. He ended his letter saying:

I’m lonely, depressed, can’t stop loving him, have problems in finding the right person, have this secret I must keep and I want to fall in love with someone. In a way, I wish he saw this letter and knew it was him, if only, if only…I would be grateful for any advice you can give me on this. Being young and gay is hell, especially when you don’t even know the bloody words for what you are. (Letter from Anonymous young man 1988)

This moving letter clearly indicates the sadness and loneliness experienced by the letter writer, and the ‘hell’ resulting from his emotional formation, growing up in a country where silences and stigma around homosexuality resulted in intense difficulty even articulating his sexuality.

The young man’s letter was published in the October 1988 issue of *Hot Press*. The reply to the problem was extremely empathetic, highlighting that his letter ‘makes powerful reading. Sad and moving and full of humanity and tenderness’. The man was advised to consider re-establishing his friendship with his former friend and to

take things easy and see how it develops. Don’t be afraid to explain to him that you are gay. […] While a sexual relationship with this guy is a possibility, it’s unlikely to work out. The closeness you enjoyed with him is something that you obviously want in your sexual relationships with men – but he may not be in a position to give you what you want sexually. That doesn’t mean he can’t be a close and valued friend to you – and vice versa (Sex Aid 1987).

The reply also went on to say ‘Being gay in our narrow-minded society can be tough but it need not be unhappy. You are lonely and depressed at the moment but there are a lot of ‘happy’ gay men and you too can establish the close relationship you are looking for in the long run’. The reply also provided details of the ‘Tel-a-Friend’ information and advice phone line for gay men so that the man could have the ‘opportunity to break the silence and talk to someone who understands and can offer a helping hand’ (Sex Aid 1987)

Letter writers as well as the readers of advice columns were also arguably part of an emotional community. Or, as David Gudelunas in his work on advice columns in the USA has suggested, ‘readers write to advice columnists to write to advice columnists to participate in a sort of public discourse, and readers who never actually mail a letter to the columnist use the column as a way to gauge their own behaviour and to eavesdrop on the problems of their friends and neighbours—many of whom they will never meet in person’ (Gudelunas 2017). The man who wrote to *Hot Press* above, expressed ‘Maybe there are others who are now feeling/experiencing what I did, or perhaps they have done so already’. While clear in expressing his loneliness and isolation, he was also aware of the wider emotional community he is part of, and the fact that other individuals reading the letter might feel the same. Advice columns of magazines provide a forum for individuals to ask for assistance with problems they are experiencing, but also, as Sue Jackson has argued in her study of Australasian teen magazines and sexual desire, can be said to ‘represent the curious interface of personal and public worlds, where personal issues, problems or concerns become available for consumption and open to scrutiny by a mass market.’ (Jackson 2005). Indeed, there was clearly an awareness among those who wrote to the advice column that if printed, their letter would be read by other young people across Ireland. It is also clear that *Hot Press* magazine was aware of the important function the Sex Aid column served in helping to consolidate an emotional community and help people to feel less alone. In his introduction to the pull-out Sex Aid supplement in March 1989, editor Niall Stokes stated ‘in bringing together some of the key topics covered in Sex Aid since its inception in this eight-page supplement, we hope not just to make people aware of the extent to which their confusion is often shared, but also to take the answers out of *their* isolation, in the conviction that read together and in continuity, a fuller and more integrated picture will emerge of the sexual issues that confront us – and how we might deal with them’ (Stokes 1989). The importance of the columns, in Stokes’ view, was clear: they not only helped young people empower themselves with knowledge around sexual health, but also provided a clearer picture of the problems facing young people in 1980s’ Ireland.

Readers themselves also alluded to the power of the problem page in providing information to young people who may have been in a similar situation to them. ‘A Worried Reader’, aged 15 years, writing to Jody at *Fresh* magazine in 1987 about her concerns about having sex with her boyfriend, added a note to the end of the letter saying ‘Please print this so that if any one else is having this problem and is afraid to write in they will know what to do with the advice you give me’. (Letter from Worried Reader n.d). Another 17-year-old, Noel, writing to Sex Aid about his incontinence problem, said ‘Please reply and print in case anyone has a similar problem’ (Letter from Noel). There was clearly an appreciation among letter writers of the power of their engagement with the column to help others as well as themselves. Similarly, the advice column helped to reassure readers that they were not alone and that there were others like them who were experiencing similar issues. Eighteen-year-old Jason, writing to Sex Aid in 1989, started his letter by saying ‘I don’t know if there are many others like me. I am eighteen and I believe I’m a transvestite. I spend as much time as I can cross-dressing in women’s clothes. As I live at home the opportunities to fully dress up are limited as you can imagine to get clothes and keep them hidden is difficult’. Jason also asked if there were any support groups available or clubs or special shops in Dublin (Letter from Jason 1989). In their reply, the advice column reassured Jason that ‘Yes, there are many other men like you who dress in women’s clothes’ and provided a detailed response outlining the characteristics of different trans identities as well as highlighting the difference between gender, fetishes and sexuality, and providing details of support lines for the Irish Transvestite Group and the Gay Switchboard (Sex Aid 1989). Such responses were clearly not only helpful to the individual writing but may have helped to reassure others with similar problems that they were not alone, while also testing the boundaries of what was acceptable to discuss in Irish society at the time.

Occasionally, authors of letters who received replies followed up again to explain how they had got on with the advice, thus entering into a dialogue with the ‘agony aunt’. Martin, for instance, wrote to Jody in September 1987 about his concern that one of his testes was smaller than the other, a problem also experienced by David mentioned earlier. He explained ‘I am very embarrassed to tell anybody’. His letter was published in the November 1987 issue and Martin was advised not to worry and ‘to put your mind at rest I’d suggest that you go along to a doctor and explain your concern. Don’t be afraid – that is what doctors are there for, and they have seen a lot of bodies in their time’ (Tears ‘n’ Fears 1987). Martin wrote to Jody to thank her for the advice in response to his query about a problem with his testicles. He explained ‘I always felt that a visit to the doctor would be the best thing but I never was able to pluck up the courage to go. I am thanking you very sincerely for your advice, it has put my mind at rest’ (Martin 1987). Martin’s letters reveal not only the relief that these advice columns provided to those who wrote to them, but also how dialogues and relationships could be built up between the author and advice giver.

## Conclusion

The agony aunt, in Tracey Loughran’s view, ‘played a crucial role in setting up the perceived bond between magazine and reader’. While the agony aunt has borne significant critique from feminist scholars for reinforcing feminine stereotypes, recent scholarship has highlighted, in Loughran’s view, ‘the importance of problem pages as windows into everyday life, social change, the authority of popular ‘experts’ and the agency of readers’ (Loughran 2020). This article, in agreement with Loughran, has illustrated the value of analysing problem pages. In the Irish case, the advice columns published in *Hot Press* and *Fresh* magazine provide the historian with a rich understanding of the types of sexual health problems which particularly affected young Irish men and women in the 1980s. The advice columns clearly served a number of functions—as well as providing young men and women with practical advice and information on a range of issues, they also served to educate young people about sexual health. More importantly, the columns were an important space for resistance against legal and religious structures which restricted young people’s access to information on sexual and reproductive health. Moreover, it is also clear that the advice columns in *Fresh* and *Hot Press* also helped to create an emotional community between readers and the ‘agony aunt’ which enabled young Irish men and women to share their problems and traverse ‘emotional frontiers’. In a country where the 1980s represented a period of silence around sex and sexual health issues, the columns allowed young people to express their feelings and analysis of the problem pages of these two magazines highlights the emotional impact that sexual health issues had on young Irish people, as well as emphasise the consequences of a society where stigma and shame around sexual issues was paramount.

An analysis of the problems received by the two magazines illustrates the impact of the lack of sex education in the country and the influence of Catholic teachings on young people’s attitudes to sex, as well as the negative emotions experienced by young people in relation to sexual health and concerns about privacy. They also illustrate the impact of restrictive legislation around contraception on young people and the pervading themes of stigma and shame. While the advice columns were not a traditional form of counselling, in an era where the Irish government fundamentally failed to provide any form of sex education or public health campaign, the advice columns became a radical act of resistance and through the straight-talking advice provided, the IFPA youth group helped to empower and encourage young people to take ownership of their sexual health, allowing them to enter into a dialogue and feel less alone.

## Data Availability

No data are available.

## Notes

1. The archive has been recently deposited at the Royal College of Physicians of Ireland Heritage Centre and is awaiting cataloguing.

2. The IFPA was founded by a group of doctors including Michael Solomons, Jim Loughran, Dermot Hourihane, Robert Towers and Joan Wilson, a social worker, Yvonne Pim, and a psychotherapist, Maire Mullarney.
